# ID 246 - Eficácia e Segurança do Transplante Intestinal e Multivisceral em Pacientes com Falência Intestinal: uma revisão sistemática

**DOI:** 10.5327/2237-9622.2025.v34s1.246

**Published:** 2025-11-25

**Authors:** Roberta Crevelário de Melo, Bruna Carolina de Araújo, Letícia Aparecida Lopes Bezerra da Silva, Cinthia Lanchotte Ferreira, Thaiana Helena Roma Santiago, Antonio Pescuma, Juliana Abud, Alessandra Crescenzi, Alexandre Chagas de Santana, Cláudia Lima Vieira, Inara Pereira da Cunha, Márcia Saldanha Kubrusly, Alex Jones Flores Cassenote, Wallace Breno Barbosa, Luciana Costa Xavier, Luciana Bertocco de Paiva Haddad

**Keywords:** transplante de intestino, transplante multivisceral, falência intestinal

## Abstract

**Introdução:**

A falência intestinal (FI) é uma condição rara, caracterizada pela incapacidade do órgão em manter a digestão e a absorção de nutrientes necessários para a manutenção nutricional do indivíduo. Estima-se que sua prevalência seja em torno de 20 a 80 casos por milhão de adultos e de 14,1 a 56 casos por milhão de crianças; além disso, que uma a três pessoas por milhão da população por ano apresentarão FI, e na população pediátrica esse número aumenta para 2 a 6,8 indivíduos por milhão. Entre a população com diagnóstico de FI, 10% a 15% serão candidatas ao transplante de intestino delgado (TID) e ao transplante multivisceral (TMV). O TID e o TMV são procedimentos indicados para pacientes com FI irreversível que correm risco de morte por causa de complicações relacionadas à nutrição parenteral prolongada e em situações de baixa qualidade de vida. Dessa forma, este estudo tem como objetivo avaliar a eficácia e a segurança de TID ou TMV comparados à nutrição parenteral prolongada em paciente com FI.

**Método:**

Foi realizada uma revisão sistemática, com base nas diretrizes metodológicas brasileiras. Realizaram-se buscas em três bases de dados: PubMed, Embase e Cochrane Library, além de consulta a especialistas, em 23 de abril de 2024. O risco de viés foi avaliado por meio das ferramentas da Cochrane e do Instituto Joanna Briggs. A confiança na evidência foi avaliada utilizando o sistema GRADE (do inglês, ).

**Resultados:**

De 804 publicações identificadas nas bases de dados, após o processo de seleção com base nos critérios de elegibilidade, foram incluídos 12 estudos, sendo 1 coorte prospectiva, 2 estudos transversais e 7 séries de casos. Os estudos apresentaram algumas falhas metodológicas quanto à clareza no relato do método.

O TID, quando comparado à dieta parenteral domiciliar, mostrou que a sobrevida em 60 meses foi superior com o transplante (RR: 0,71; IC de 95%: 0,53 a 0,96; p=0,02). Contudo, outro estudo, realizado em nove meses, não encontrou diferença significativa na qualidade de vida entre os grupos, exceto para dor corporal (DM: 0,96; IC de 95%: 0,18 a 1,74; p=0,02). A sobrevida dos pacientes após TID variou entre 60% e 100% em 2 a 108 meses, e a sobrevida do enxerto variou entre 25% e 82% em 2 a 60 meses. A rejeição aguda variou de 22% a 88% em 1 a 12 meses, e a rejeição crônica de 0% a 29% em 1 a 48 meses. O retransplante foi necessário em 3% dos pacientes em 84 meses, e complicações imunológicas ocorreram em 29% dos casos. Não houve ocorrência de doença do enxerto contra hospedeiro (DECH).

O TMV apresentou sobrevida dos pacientes de 57% em 12 meses, variando de 27% a 67% em 60 meses. A sobrevida do enxerto foi de 42% a 57%, em 12 meses, e de 27% a 61% em 60 meses. A descontinuação da NP ocorreu em 60% dos pacientes em 13 meses. A rejeição aguda foi de 66% em 1 mês e de 42% em 48 meses, sem relatos de rejeição crônica. O retransplante foi necessário em 9% dos pacientes em 84 meses, e complicações imunológicas variaram de 27% a 50%. Não houve ocorrência de DECH.

**Conclusão:**

Embora haja escassez de estudos na pirâmide da evidência na literatura científica que comprovem a eficácia e a segurança dos transplantes, especialmente pela indicação de tratamento para condições ultrarraras, TID e TMV, quando bem indicados, mostram melhor sobrevida do paciente com FI que tem impossibilidade de continuar na nutrição parenteral prolongada.

